# Different Effects of Perceived Social Support on the Relationship between Perceived Stress and Depression among University Students with Borderline Personality Disorder Symptoms: A Multigroup Mediation Analysis

**DOI:** 10.3390/healthcare10112212

**Published:** 2022-11-03

**Authors:** Narusorn Ingkachotivanich, Tinakon Wongpakaran, Nahathai Wongpakaran, Awirut Oon-Arom, Nuntaporn Karawekpanyawong, Trustsavin Lohanan, Thanakorn Leesawat

**Affiliations:** 1 Department of Psychiatry, Faculty of Medicine, Chiang Mai University, Chiang Mai 50200, Thailand; 2 Faculty of Medicine, Chiang Mai University, Chiang Mai 50200, Thailand

**Keywords:** borderline personality, family support, friends support, depressive symptoms, mediation

## Abstract

Background: While perceived social support can mediate the relationship between perceived stress and depression, little is known about the differences between individuals with high and low borderline personality disorder symptoms (BPDS). This study aimed to investigate the associations among perceived stress, perceived social support, and depression, and compare low and high levels of BPDS. Methods. This cross-sectional analysis was a secondary analysis of data from the SI-Bord study. University students across Thailand completed a screening instrument for borderline personality disorder, the Perceived Stress Scale (PSS), the Revised Thai Multi-dimensional Scales of Perceived Social Support (MSPSS), and the Patient-Health Questionnaire (PHQ)-9. Mediation analysis using PROCESS was applied to test the direct and indirect effects of perceived stress on depression. Multigroup mediational analysis was adopted to compare low and high levels of BPDS. Results. The mean age of the 330 participants was 20.27 (SD, 1.4) and 80% were female. Significant correlations were observed between the PSS, MSPSS, and PHQ scores, with greater magnitude among the high-level BPDS group (*p* < 0.001). A significant direct effect on perceived stress and a significant indirect effect on depression through perceived social support were noted. Of all the sources of social support, only the significant others variable significantly differed between the two groups (*p* < 0.05). Conclusion. Perception of social support had a significant mediating role in perceived stress and depression. The magnitude of associations was remarkably high for individuals with high BPDS compared to those with low BPDS. Unlike those with low BPDS, all sources of social support were significant mediators between the two groups.

## 1. Introduction

Depression is considered an important and commonly found public health problem that leads to suicide. Evidence confirms that depression affects more than 264 million people worldwide [1]. College and university students are at risk of experiencing stress due to a variety of factors, such as pressure to study, exams, inadequate rest time, inappropriate exercise and food intake, competition, coping with family expectations, economic status, chronic illness, and use of alcohol, cigarettes or other substances [2,3,4,5]. Depression causes psychological distress, may negatively affect academic performance [6], and can result in poor quality of life [7,8,9]. The prevalence of university students reporting depression ranges from 10 to 85%, with a weighted mean of 30.6% [10], and on average, a prevalence rate of 24.4% is reported in low and middle-income countries [11].

The severest consequence of depression is suicidality. Like other populations experiencing depression, the prevalence estimates for suicidal ideation for university students range from 9.7 to 58.3%, with a 27.1% prevalence for suicidal ideation in life, 14.1% for suicidal ideation in the last year, 3.1% for attempted suicide in life [12], and 7.4 to 24.2% observed among medical students [13]. Many factors are associated with suicidality, one of which is borderline personality disorder (BPD) [14,15,16,17].

BPD is a serious mental illness characterized by the insecurity of identity and life goals, interpersonal relationships, lack of restraint, risk-taking, and emotional instability. Related studies have documented the relationship between depression and borderline personality disorder symptoms (BPDS). Insecure attachment and persistent feelings of emptiness render a feeling of depression among individuals with BPDS [18]. Over 80% of individuals with BPD experience depression leading to self-harm or suicide at least once in their lives [19].

In addition to depression, individuals with BPD are vulnerable to stress. Apart from childhood psychological trauma and a poor sense of self-ascribing sensitivity to stress, BPD has been shown to have an underlying biological abnormality. For example, patients with BPD may show a neurovegetative imbalance [20,21] and altered hypothalamus–pituitary–adrenal axis functioning [22,23]. All the abnormal biopsychological underpinnings render individuals with BPD to be liable to stress. 

An inability to cope with stress could lead to depression. In other words, stress is usually perceived as a herald of depression. Perceived stress is strongly related to depression across all age ranges [24,25,26], including for university students [8,9]. It has also been well-established that high levels of stress are associated with high levels of depression [27,28,29,30]. 

Along the path between stress and depression, many intervening variables exist, such as coping patterns [31], particularly perceived social support [26,32]. Social support plays an important role in reducing depressive symptoms. Social support refers to the mechanisms by which interpersonal relationships presumably buffer individuals against a stressful environment [33], and is defined as the care, support, and assistance received from families, friends, and communities [34]. 

An effective social support system could protect adolescents and young adults from interpersonal life stress and psychological distress [35,36], and loneliness [37]. Students who perceive that they have good social support, especially from family members and friends, encounter a lower incidence and severity of depression than students who do not receive good social support [38,39,40,41,42,43,44]. 

The role of perceived social support as a mediator in the relationship between stress and depression has been widely studied amongst a variety of stressors [45,46,47,48] and in a variety of populations. For example, postpartum women [49], women with breast cancer [50], university graduates [51], and pregnant women [52] have all confirmed that perceived social support mediates the relationship between perceived stress and depression.

What we have yet to discover is whether these relationships exist among individuals with borderline personality disorder. To investigate this issue, the authors explored a mediation model between two groups of university students, one with low and another with high BPDS; this is because individuals with borderline personality symptoms tend to have more stress and depression, and less social support. We hypothesized that the correlation between these three variables would be stronger in the group with high-level BPDS than in the group with low-level BPDS.

## 2. Materials and Methods

### 2.1. Study Design

This research constituted a secondary analysis of data from the project entitled, “Development and validation of a screening instrument for BPD (SI-Bord) for use among university students” [53], which was conducted among Thai students from November to December 2019. As this study comprised a secondary analysis, ethics exemption was granted by the Ethics Committee, Faculty of Medicine, Chiang Mai University, Thailand. The original research was approved by the Institutional Review Board (or Ethics Committee) of the Faculty of Medicine, Chiang Mai University (study code, 365/2562; date of approval, 31 October 2019).

### 2.2. Sample Size Calculation

We calculated the sample size for the mediation analysis using Monte Carlo Power Analysis for Indirect Effects [54]. The correlation coefficient (r) between variables was used for calculation. The previous study showed that between the PSS and PHQ-9, r was 0.722; between the PSS and MSPSS, r was −0.458; and between the MSPSS and PHQ-9, r was −0.520 [53]. The calculations yielded a sample size of 83 to exhibit a statistical power of 80%, whereas a sample size of 104 exhibited a power of 90%. Therefore, the total sample size of 330 participants in this study was promising.

### 2.3. Participants and Setting

**Figure 1:**
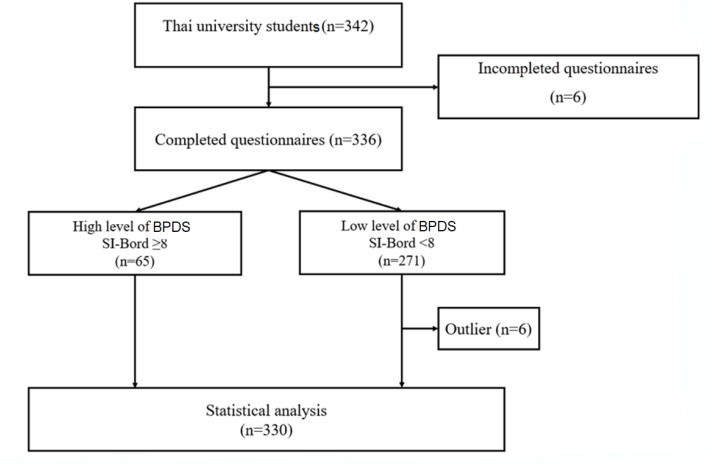
Flow chart for the study sample size. BPDS = borderline personality disorder symptoms. SI-Bord = screening instrument for borderline personality disorder.

All the stored data from 342 participants were initially used. These consisted of university students residing in Thailand during 2019, aged from 18 to 25 years. The exclusion criteria included those with a diagnosis of psychiatric disorders such as schizophrenia, bipolar disorder, drug or alcohol use disorder, and a record of alcohol use within 24 h of participating in the research. Figure 1 shows that only 330 participants were used for the final analysis.

### 2.4. Measurements

#### 2.4.1. Screening Instrument for BPD (SI-Bord)

SI-Bord is a self-reported screening tool that assesses BPD. It consists of 5 questions, scored as 0 to 3 (0 = not at all, 1 = slightly, 2 = moderate, 3 = mostly). A high SI-Bord score indicates a high level of BPD symptoms. This research used SI-Bord ≥8, yielding a sensitivity of 75.00% (95% CI 47.6–92.7%) and specificity of 73.08% (95% CI 59.0–84.4%), to determine whether the respondent had a high level of BPDS [53]. The total scores ranged from 0 to 15, and higher scores indicated more BPD symptoms or traits. Cronbach’s alpha for the SI-Bord was 0.76.

#### 2.4.2. 10-Item Perceived Stress Scale (PSS-10)

The PSS-10 is a self-reporting questionnaire that measures stress level. It consists of 10 questions, with a 5-point Likert scale ranging from never (0) to very often (4). Higher total scores denote higher levels of stress [55]. The Thai version of the PSS-10 demonstrates good psychometric properties [56], and a Cronbach’s alpha of 0.85 was demonstrated in the present sample.

#### 2.4.3. Revised Thai Multi-Dimensional Scales of Perceived Social Support (r-MSPSS)

This tool is a multidimensional feeling questionnaire about three sources of social support, i.e., social support from family members, friends, and significant others (or special individuals). It consists of 12 questions, with a 7-point Likert scale ranging from very strongly disagree (0) to very strongly agree (6). Higher scores reflect higher levels of perceived social support. The Thai version demonstrated good psychometric properties [57]. The current sample exhibited a Cronbach’s alpha of 0.91.

#### 2.4.4. Patient-Health Questionaire-9 (PHQ-9)

The PHQ-9 is a 9-item self-reporting questionnaire measuring level of depression. It consists of 9 questions with a 4-response Likert scale ranging from 0 (not at all) to 3 (nearly every day). Higher total scores indicate higher levels of depressive symptoms [58]. The Thai version of the PHQ-9 showed a Cronbach’s alpha of 0.79 and a significant association between the PHQ-9 and the HAM-D [59]. A Cronbach’s alpha of 0.89 was obtained for the current sample.

### 2.5. Statistical Analysis

Descriptive statistics were used for the demographic data, e.g., age and sex. Pearson’s correlation was used to find the relationship between the SI-Bord, PSS-10, r-MSPSS, and PHQ-9 scores. The hypothesized mediation model tested all significantly correlated variables to see how perceived social support influences the relationship between perceived stress and depression. The indirect effect of perceived stress on depression through perceived social support was tested. For the mediation analysis, the researchers used methods discussed by Hayes [60] to examine the relationship between perceived stress (X) and depression (Y) through perceived social support (M1) (Figures 3 and 4).

A mediation model was analyzed using IBM SPSS 22 and macro-PROCESS, Version 4.0 [61]. As suggested when conducting mediation analysis, 5000 bootstrap resampling and the product of coefficients were performed [50]. Unstandardized regression coefficients (B) and *p*-values were reported for the direct effect coefficients and bootstrap confidence intervals were reported for conditional indirect effects. Confidence intervals that did not straddle zero were indicative of statistical significance. Multigroup mediation analysis was used to compare the mediation model between groups with or without BPD symptoms using Amos, Version 18 (IBM Corp., Armonk, NY, USA). Critical ratios for differences between pairwise parameters were calculated. MedCalc Version 19.7 (MedCalc Software, Mariakerke, Belgium) was used to create the graphs. For all the analyses, the level of significance was set at *p* < 0.05.

## 3. Results

The subjects were mostly young females; the number of years of studying ranged from 1 to 6 years. Significant differences in age, sex, and years of studying were found between the two groups (Table 1).

**Figure 2:**
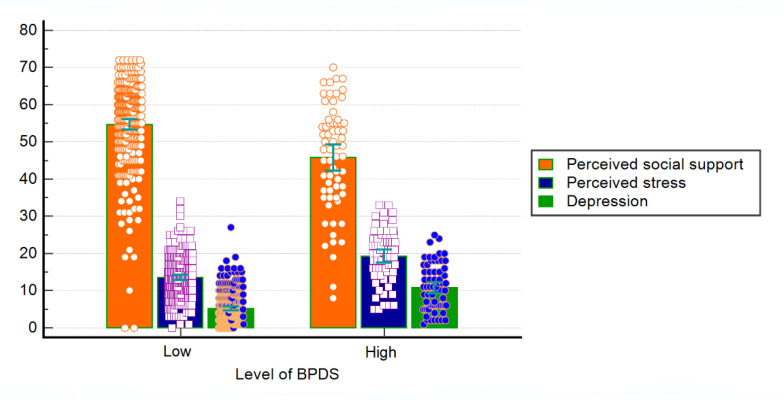
Clustered bar graphs illustrating dots, means, and confidence intervals for each group using a bar chart with error bars. BPDS = borderline personality disorder symptoms.

Figure 2 shows that perceived stress and depression were higher for the high-level BPDS group; on the contrary, the mean score for perceived social support was lower for the high-level BPDS group (all *p* < 0.001) 

The correlation coefficients between the PSS, MSPSS, and PHQ scores were −0.459, 0.721, and −0.524 (all *p* < 0.01), for the PSS and MSPSS, PSS and PHQ, and MSPSS and PHQ, respectively. These correlation coefficients varied according to high and low BPDS levels. The magnitude of correlation was greater for the high-level BPDS group compared to the low-level BPDS group, as shown in Table 2.

**Figure 3:**
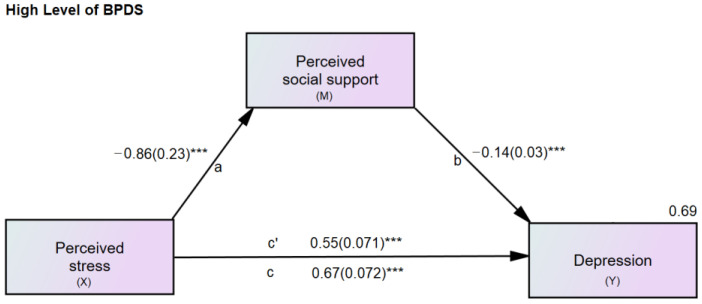
Mediation model of perceived stress, social support, and depression for high levels of borderline personality symptoms. X = predictor; M = mediator; Y = outcome; a, b, c, c′ = path coefficients; c = total direct effect of perceived stress on depression; c′ = direct effect of perceived stress on depression controlling for perceived social support. Values for depression (Y) are the R-square. BPDS = borderline personality disorder symptoms. *** *p* < 0.001.

**Figure 4:**
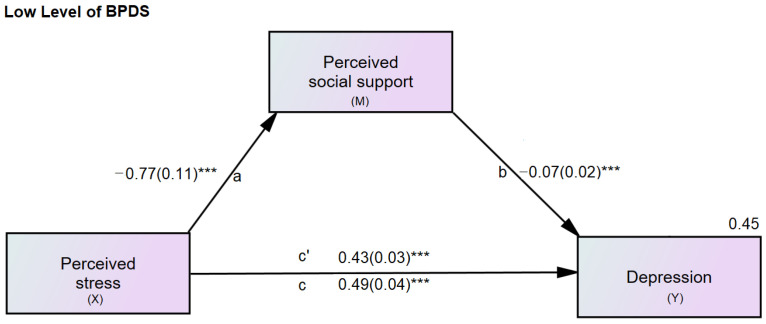
Mediation model of perceived stress, social support, and depression for low levels of borderline personality symptoms. X = predictor; M = mediator; Y = outcome; a, b, c, c′ = path coefficients; c = total direct effect of perceived stress on depression; c′ = direct effect of perceived stress on depression controlling for perceived social support. Values for depression (Y) are the R-square. BPDS = borderline personality disorder symptoms. *** *p* < 0.001.

Figure 3 and Figure 4 show the unstandardized estimation coefficients of the direct effects of perceived stress on depression and perceived social support, and the direct effect of perceived social support on depression. All path coefficients were significant (*p* < 0.001). The direct effect of perceived stress was reduced from B = 0.67 (c) to B = 0.55 (c′), controlling for the mediator, perceived social support. The variance for depression explained by this model was 69% for the high-level BPDS group. The same was true for low levels of BPDS; the direct effect of perceived stress was reduced from B = 0.49 (c) to B = 0.43 (c′), controlling for the mediator, perceived social support. The variance for depression explained by this model was 45%.

In the mediation analyses for each subscale of social support, i.e., significant others, family members, and friends, a similar pattern of results was found, except for the indirect effect of the model where significant others served as a mediator (B = 0.016, 95%CI = −0.001 to 0.043) (Table 3).

Comparing the path coefficients (B) between high and low levels of BPDS using multigroup analysis identified no significant critical ratios for family and friends. Only the b path (the path between significant others and depression) was significant (*p* < 0.05) (Table 4).

## 4. Discussion

This study aimed to investigate how perceived social support mediated the relationship between perceived stress and depressive symptoms when comparing two groups with high and low levels of BPDS. The main findings demonstrated that perceived social support had a significant mediating role for perceived stress and depressive symptoms. Furthermore, the magnitude of such associations was more remarkable in the high-level BPDS group than in the low-level BPDS group. These findings support our hypothesis that perceived stress and support significantly affect depression among individuals with borderline symptoms. Furthermore, regarding the specific source of social support, all were significant mediators in the high-level BPDS group.

In contrast, only friends and family members showed significance in the low-level BPDS group. These findings were confirmed by multigroup analysis, which revealed that only the mediation model, where significant others served as a mediator, differed significantly between the two groups. The results highlight the crucial role of significant others or special people for individuals with BPDS.

The mechanism by which social support is associated with decreased depression among individuals with BPDS can be explained through attachment theory [62]. Researchers have demonstrated that individuals with borderline personality disorder symptoms are likely to have preoccupied or fearful insecurity and hypersensitive reactions, making them less effective at handling interpersonal stress [63]. This evidence was notably demonstrated during the COVID-19 pandemic, where physical and social distancing measures were applied [64]. Such feelings of insecurity and loneliness render these individuals longing for support and emotional and social closeness. However, some individuals, especially those with fearful attachment issues, are usually reluctant to seek support from others for fear of rejection. This is based on the fact that high-level BPDS is attributed to insecure attachment derived from unfulfilled childhood experiences [65], resulting in an inability to effectively cope with stress and a striving for emotional support from surrounding people [66]. Significant others are inevitably an additional source of emotional support as family members or friends cannot usually fulfill all their emotional needs. These special people usually include seniors at university, professors, or even health care providers [67,68], who are especially sought out when individuals with high-level BPDS experience more severe depression or suicidality. Of all the personality disorders, it is evident that individuals with borderline personality disorder predominantly seek help for their major depression [17,69,70].

Another reason why special individuals are essential for students with BPDS could be because such students might be unable to obtain full support from family members due to pre-existing conflicts [65,66]. Indeed, a predisposing factor in the development of borderline personality is unempathetic parents, which leads to a feeling of insufficient support from family members. Moreover, individuals with BPDS tend to have social inhibition and poor interpersonal skills [71,72]. This makes it difficult for them to reach out to their friends for help when needed. Therefore, seeking support from special people besides family members and friends seems inevitable, whether it is actively sought out or the meeting is enforced due to severe depression or suicidality.

Related studies indicate that perceived social support tends to be negatively associated with psychiatric disorders, especially depression [38,73,74]. Regarding the source of social support, the findings of this study support other studies regarding the importance of friends and family members among young people. For example, Hadebe and Ramukumba have demonstrated that young adults who live with mental illness and enjoy support from family members and friends can cope with stressful challenges and have a better outlook for the future [43].

Inconsistent findings have been found in other related studies. For example, while the present study demonstrated that BPDS is related to the male sex, many studies have found no sex differences [75,76]. Some studies, however, have found a higher prevalence in the female sex [77,78]. In addition, the present study found that older age groups and more years of studying were related to BPDS, in contrast to some studies with younger age groups [78]. Methodological or study sample factors may be responsible for these discrepancies. Therefore, to be able to reliably compare these studies, the same measures would have needed to be used across all the studies.

### 4.1. Implications and Future Research

Identifying BPDS among university students is essential so that an appropriate plan, which includes sufficient social support, primarily from additional sources, besides friends and family members, can be provided. Furthermore, interventions to promote positive attributes and coping skills should be proactively implemented, especially for those with a high BPDS level, to prevent severe depression or suicidality. In addition, when an individual with a high level of borderline symptoms is identified, a support system, regardless of source, should be identified in a timely fashion. Both sides, university students and health care providers, would benefit from further research on training students to effectively access support, and training health care personnel and faculty counselors to effectively provide for students with BPDS.

### 4.2. Strengths and Limitations

To the best of our knowledge, this study constitutes one of the first to demonstrate the beneficial role of social support for depression among those with BPDS. However, the research has some limitations that should be addressed. Firstly, the groups were categorized using the SI-Bord cut-off criteria instead of clinical diagnosis, which can identify false-positive cases; inevitably, it was not possible to identify these types of cases in research based on an online survey. Secondly, the cross-sectional design precluded making robust conclusions regarding any causal relationship. Therefore, longitudinal mediation analysis studies are warranted. Thirdly, this study was conducted in Thailand and cultural factors might have influenced the outcomes, especially those related to social support. Therefore, replication studies in other countries are warranted. Finally, the findings cannot be generalized to all Thai university students due to the nonrandom sampling method that was used.

## 5. Conclusions

The present study has demonstrated that perceptions of social support, especially from significant or special people, are essential for students with BPDS. Furthermore, the relationships between perceived social support and stress, and between social support and depression are more extraordinary among those with high-level BPDS than they are among those with low-level BPDS. This novel evidence provides a guide that can help young adults to deal with depression. Future research on identifying borderline personality disorder symptoms and early social support interventions should be encouraged.

## Figures and Tables

**Table 1:** Participants’ characteristics (*n* = 330).

Variable	All (*n* = 330)	High Level of BPDS (*n* = 65)	Low Level of BPDS (*n* = 265)	Test Difference
Age	20.27 ± 1.4	21.05 ± 1.4	20.08 ± 1.3	*t* = 5.23, *p* < 0.001
Sex, female	264 (80.0%)	43 (66.2%)	221 (83.4%)	*t* = 9.70, *p* < 0.001
Years of studying, median (interquartile range)	3(2)	4(2)	2 (2)	χ^2^(1) = 31.844, *p* < 0.001

BPDS = borderline personality disorder symptoms.

**Table 2:** Comparison of the correlation coefficients between low levels of BPDS and high levels of BPDS.

	PSS	MSPSS	PHQ
PSS	-	−0.395 **	0.645 **
MSPSS	−0.428 **	-	−0.421 **
PHQ	0.764 **	−0.589 **	-

The correlation coefficients among low levels of BPDS (n = 265) are in the upper quadrant; the correlation coefficients among high levels of BPDS are in the lower quadrant (n = 65). PSS = Perceived Stress Scale, MSPSS = Multidimensional Scale of Perceived Social Support, PHQ = Patient Health Questionnaire, BPDS = borderline personality disorder symptoms. ** *p* < 0.01.

**Table 3:** Summary of the indirect and indirect effects of the mediation models, adjusted for age, sex, and level of education.

Relationships Perceived Stress → Perceived Social Support → Depression	Level of BPDS	Direct Effect (B) (*t*-Statistics)	Indirect Effect (B)	Confidence Interval		*R^2^*
				Lower Bound	Higher Bound	
Total	Low	0.428(11.38) **	0.056 **	0.026	0.114	0.454
	High	0.553 (7.80) ***	0.120 **	0.041	0.240	0.685
SO	Low	0.468 (13.27) **	0.016	−0.001	0.043	0.429
	High	0.606 (8.98) ***	0.067 **	0.005	0.167	0.667
FM	Low	0.430(11.20) **	0.055 **	0.016	0.111	0.449
	High	0.568 (7.79) ***	0.105 **	0.037	0.204	0.667
FR	Low	0.442(11.80) **	0.043 **	0.010	0.090	0.446
	High	0.599 (8.06) ***	0.074 **	0.011	0.184	0.641

Note: Bootstrap sample = 5000 with replacement. BPDS = borderline personality disorder symptoms, SO = significant others, FM = family, FR = friends. *** *p* < 0.001, ** *p* < 0.01.

**Table 4:** Critical ratios for differences between pairwise parameters.

Pairwise Comparison	Total	Significant Others	Family	Friends
a1-a2	0.304	0.484	−0.069	0.252
b1-b2	1.641	2.278 *	0.957	0.899
c1-c2	−1.521	−1.697	−1.824	−1.837

* *p* < 0.05.

## Data Availability

The datasets used and/or analyzed during the current study are available from the corresponding author upon reasonable request.
